# Physiology and Pathology of First Dentition

**Published:** 1886-08

**Authors:** H. D. Eggers

**Affiliations:** Louisville


### THE DENTAL REGISTER.

Vol. XL.] AUGUST, 1886. [No. 8.

### Communications.

### Physiology and Pathology of First Dentition.

BY H. D. EGGERS, LOUISVILLE.

Of all the functions of the animal economy none are more curious or interesting than those concerned in the production of the teeth. The formation of the teeth in the fœtus begins at a very early period, about the seventh week. On the surface of the jaw there is found a depression or groove, the surface of which is formed of epithelial cells, the tissue underneath this is of a gelatinous and cellular substance, and below this is the ossifying substance of the jaw. The essential structures of the teeth are derived from these two elements, the enamel from the epithelium which covers the surface of the dental groove, the dentine and crusta petrosa from the deeper structures.

First, as to the formation of enamal. The epithelium becomes thickened and protrudes over the margins of the dental groove, and then penetrates down into it, and as the sides of the groove rise up, the epithelial mass (or "enamel organ") seems to pass deeper and deeper into the substance of the jaw, meeting with the papilla, from which the dentine or bulk of the tooth is developed, forming the shape of a cap, which unites with the superficial layer of epithelium by a neck or string of epithelium. This string is the future enamal organ of the permanent tooth. The enamel organ folds itself over the dental papillæ as it grows up from the bottom of the dental groove, still epithelial, presenting an outer and inner surface and an intermediate portion changing into the proper enamel tissue.

The covering of epithelium on the outer surface of the enamel long remains perceptible. After the tooth has cut through the gum, this layer of epithelium may be removed from the tooth, in form of a membrane by the action of strong acids. This membrane, which is the cuticula dentis or Nasmyth's membrane, is marked by the hexagonal impress of the enamel prisms, and if stained by nitrate of silver will show the characteristic appearance of epithelium. This membrane, however, soon wears away from the surface of the tooth.

The calcification of the epithelial cells forms the bulk of the enamel, which cells are changed into hexagonal prisms, the anastomosing of which with each other, forms the hexagonal rods of the matured enamel.

About this time a projection of the mucous tissue of the infantile jaw springs from each side of the base of the pulp, and grows toward the summit of the tooth, where it meets with that of the opposite side, and so forms the dental sac.

The next step is the formation of the odontablasts, which have a relation to the development of the teeth similar to that of the osteoblast to the formation of bone. These are large neucleated cells of elongated form and provided with numerous processes developed from the cells of the dental papilla. The odontoblasts send out processes, which as they grow, become calcified externally, the calcified portion forming the dentine, the uncalcified part the dentinal fibres.

The cement is ordinary bone, containing canaliculi and lamiæ, and is developed from the deeper tissues of the fœtal jaw, exactly as bone is produced in other parts of the body by periosteal ossification. Haversian canals are sometimes found where the cement is thick. The germs of the decidous teeth make their appearance in the following order:

The germ of first superior molar appears at the seventh week, at the eighth week the canine. The central incisors at the ninth week. Lastly, the second molar is seen at the tenth week.

When the calcifications of the different tissues of the tooth are sufficiently advanced to enable it to bear the pressure to which it will be afterwards subjected, its eruption takes place, the tooth making its way through the gums.

PATHOLOGY.

The evolution of the teeth is usually attended by more or less turgescence around the dental bulbs or follicles, greater with some teeth than with others. The superior teeth cause more swelling than do the inferior incisors.

This swelling, although attended by more or less conjestion, is physiological within certain limits, and not a disease. But sometimes there is an unusual amount of swelling around the dental follicles, the afflux of blood to them is greatly increased; they are the seat of such a degree of tenderness and pain that the infant is fretful. The infant will indicate the seat of its suffering by carrying the finger to its mouth. These pathological results of dentition though they may interfere more or less with feeding or sleeping are not dangerous. They are easily detected, and are caused directly from the rapid growth and increased sensibility of the dental follicles.

Some children before the eruption of the teeth are affected with diarrhœa accompanied by irritability of the stomach. Some writers claim that in these cases, gastro-intestinal catarrh is present, others, that there is simply a hypersecretion, in other words, one of the forms of non-inflammatory diarrhœa. Barrier believes that in occasional cases it is due to defective or altered innervation. It would then be analogus to that form of diarrhœa which occurs in the adult from the emotions. It is certain, however, that in most cases of diarrhœa which are attributed to dentition, there are other causes, such as unhealthy localities, bad food, uncleanliness, lack of attention. Especially in city infants the chief causes of diarrhœa during the period of dentition are strictly anti-hygienic.

But when, as sometimes happens, at each period of dental evolution, the child is affected with diarrhœa, then the influence of teething is apparent.

Convulsions are a common pathological result of difficult dentition, and are due in most cases to excitement of the nervous system arising from the pain, and to a determination of blood to the dental apparatus, in which the whole vascular system of the head participates. A careful examination in some of these cases, however, will lead to the detection of other causes in addition to the state of the gums.

Convulsions in exceptional cases occur mainly from dentition. This may happen when several teeth erupt at about the same time.

Teething less frequently disturbs the respiratory organs than either the digestive or cerebro-spinal system. In some infants a cough occurs at each period of dental evolution with slight expectorations. In some cases a high pulse is often observed at the time of great swelling and tenderness which subsides with protrusion of the tooth.

Cutaneous diseases are common during dentition but their dependence on it as a cause is not clearly demonstrated.

Diagnosis. The abnormal symptoms which occur during the period of dentition are easily diagnosticated. When similar symptoms occur at each period of teething, and subside with the subsidence of the swelling and tenderness, teething must be regarded as the cause, or if the disease be such as is known to be produced by difficult teething, and after careful examination fail to find other causes, while the gums being swollen and tender, it is proper to attribute the trouble to dentition. As a rule, however, if there be any other cause apparent the influence of dentition may be regarded as a matter of secondary consideration.

Prognosis in the majority of cases is favorable.

### TREATMENT.

The remedies to be used in difficult dentition must necessarily be two-fold, namely, local and constitutional. Local, those that are fitted to diminish the swelling and tenderness of the gums. Constitutional, those that check the copious discharge of the bowels. In nervous affections, soothing remedies that will relieve pain and produce rest. Bromide of potassium ranks high as a useful and safe remedy in fretfulness and nervous excitement.

A few more words in regard to the use of the lancet in difficult dentition and I shall have finished.

The gum lancet is not as much used now as it was formerly. Lancing the gum over an ordinary tooth does not relieve the tooth as many seem to think, nor is the gum rendered tense by pressure of the tooth; if it were, the edges of the wound would stand apart and not heal up by first intention in the first few days, as is generally the case, unless indeed the tooth is on the point of protrusion.

If there be no other symptoms except those that occur from a swollen and conjested state of the gum the lancet should be seldom used. The only abnormal condition of the gum in which the lancet should be used is an abscess over the coming tooth. Should the patient be in danger, as in convulsions, and all other remedies fail to speedily afford relief, it may then be proper to resort to the lancet, for in such cases all measures, provided they be safe and simple, should be employed without delay.

